# Patients’ experiences of Parkinson’s disease: a qualitative study in glucocerebrosidase and idiopathic Parkinson’s disease

**DOI:** 10.1186/s41687-020-00230-9

**Published:** 2020-08-05

**Authors:** N. Bonner, S. Bozzi, L. Morgan, B. Mason, M. J. Peterschmitt, T. Z. Fischer, R. Arbuckle, M. Reaney

**Affiliations:** 1 Adelphi Values, Manchester, UK; 2 Sanofi, Paris, France; 3 Sanofi-Genzyme, Cambridge, USA; 4 IQVIA, Reading, UK

**Keywords:** Parkinson’s disease, Glucocerebrosidase mutation, Qualitative interviews, Patient experience

## Abstract

**Background:**

Approximately 7–10% of Parkinson’s disease (PD) patients carry a GBA (Glucocerebrosidase) mutation (GBA-PD patients), which may influence the disease’s clinical course.

**Objectives:**

This study aimed to explore the patient experience of GBA-PD and identify the most important symptoms and impacts to inform clinical trial measurement strategies.

**Methods:**

Twenty PD patients (*n* = 15 GBA-PD; *n* = 5 idiopathic-PD) participated in qualitative interviews which explored concepts spontaneously reported or identified through a literature review. Telephone interviews with five expert clinicians included discussion of a preliminary conceptual model derived from literature. Verbatim transcripts were thematically analysed.

**Results:**

Thirty symptoms reported by patients were categorized as motor, non-motor, and cognitive/psychiatric. Tremor (*n* = 13), memory loss (n = 13), rigidity/stiffness (*n* = 11), and speech problems (n = 11) were considered the most important and impactful symptoms by GBA-PD patients, although other symptoms were also relevant to the majority of patients. Key impacts included: sleep disturbances (*n* = 13), handwriting changes (n = 13), reduced social interaction (*n* = 12), dyskinesia (*n* = 10), depressed mood (*n* = 9), and fear of falling (*n* = 8). Key symptoms and impacts reported by GBA-PD patients were consistent with those reported by idiopathic-PD patients. Clinician interview results supported the patient findings, although some clinicians indicated that cognitive/psychiatric symptoms may present earlier in GBA-PD patients. The concepts emerging from the research informed updates to a conceptual model of GBA-PD patients’ disease experience.

**Conclusions:**

The findings provide in-depth understanding of the patient experience of GBA-PD. The findings confirm that the concepts relevant to assess in GBA-PD are consistent with those relevant to assess in idiopathic-PD; however, greater consideration of cognitive/psychiatric symptoms may be warranted in GBA-PD populations.

## Introduction

Parkinson’s Disease (PD) is the second most common neurodegenerative disorder after Alzheimer’s disease and affects about 0.1 to 0.2% of the general population and 1% of the population over the age of 65 [1, 2]. This progressive neurodegenerative condition is characterized by three cardinal motor symptoms: resting tremor, bradykinesia and rigidity; however, non-motor symptoms are also common, including cognitive impairment, hallucinations, autonomic and sensory dysfunction and sleep disorders [3, 4]. Based on existing qualitative literature about the experience of patients with idiopathic-PD (iPD), both motor and non-motor symptoms independently determine the course of the disease, the impact on quality of life and PD-associated mortality [5].

Approximately 7–10% of PD patients possess a glucocerebrosidase mutation (GBA-PD) [6]. There is some evidence that GBA mutations may influence the clinical course and phenotypic expression of PD, specifically leading to more rapid progression of cognitive/psychiatric symptoms [2, 4, 7]. An unpublished review of published literature and regulatory guidance documents revealed details of the patient populations, key signs/symptoms of GBA-PD (including subcategories of motor-functioning, cognitive & psychiatric, and non-motor functioning), and impacts of GBA-PD (including eating abnormalities, falls and damage to bone structure, sleep problems, hospital admissions, physical functioning, role functioning, emotional functioning, and social functioning). This literature was used to support the development of a draft conceptual disease model of GBA-PD, which detailed the concepts associated with GBA-PD, and included links indicating causal relationships between them. Although the literature review provided an indication of the signs and symptoms of GBA-PD, and various qualitative studies in iPD have previously been conducted [5, 8], there is a dearth of qualitative data relating to the patient experience of GBA-PD.

This study aimed to increase understanding of the patient experience of GBA-PD via the conduct of exploratory, qualitative concept elicitation interviews with both GBA-PD expert clinicians and patients to provide a better understanding of GBA-PD and inform revisions to the conceptual disease model.

## Methods

This was a non-interventional, qualitative interview study with 20 US English speaking individuals with PD and five clinical experts (neurologists and clinical geneticists), who were actively involved in the treatment of patients with GBA-PD. The study was conducted in accordance with the Declaration of Helsinki and approved and overseen by Copernicus Group Independent Review Board (IRB), a centralized review board in the US (reference: ADE1–17-542). Patients and clinicians provided written informed consent prior to any study activities including the collection of any of their data.

### Study sample

Originally, it was intended to interview 20 GBA-PD patients; however, recruitment of this subpopulation proved difficult. As a result the sample of 15 GBA-PD patients was supplemented with five iPD patients. The small iPD sample size prevents strong cross-population comparisons; however, the inclusion of an iPD sample allows for reference to be made GBA-PD between the GBA-PD and wider iPD populations.

Therefore, a total of 15 GBA-PD patients, five iPD patients, and five expert clinicians participated in two-hour, semi-structured qualitative interviews. Both clinical expert and patient participants were recruited according to pre-defined inclusion and exclusion criteria. In particular these criteria specified that all patients had to be over the age of 18 (with no upper age limit) and have a clinician-confirmed diagnosis of GBA-PD or (following the modification of the inclusion criteria) iPD and had to be willing and able to participate in the interview. The clinician criteria specified that clinician participants were required to be currently treating GBA-PD patients. Quotas were applied to the patient sample to ensure a diverse sample with representation of a range of demographic (e.g. sex, age) and clinical (e.g. stage of condition as defined by Hoehn and Yahr score) characteristics. The clinical experts (none of whom were members of the study team or are authors on this paper) were identified by the study sponsor to reduce delays and ensure clinicians with adequate experience were recruited. Patient participants were recruited through clinician referrals.

### Interview methodology

Semi-structured interview guides were used to guide both the expert clinician and patient interviews to ensure that all topics of interest were discussed. The guides were developed based on the unpublished review of literature in iPD and GBA-PD and a selection of the open-ended questioning from the patient interview guide is included in Table 1. Five clinicians took part in two-hour long qualitative interviews conducted by telephone by a trained interviewer. Clinicians were first asked open-ended questions about the patient experience of GBA-PD symptoms and impacts and how this relates to the clinical characteristics of the disease. Moreover, the clinicians were asked whether there were any differences in the relevance or manifestation of the symptoms and impacts between GBA-PD and iPD patients. During the interview, clinicians were given, reviewed, and provided feedback on a draft GBA-PD conceptual model, created based on a preliminary literature review that is outside of the scope of this article. Clinicians were provided with the conceptual model during the interview to ensure that the model did not influence initial discussions about the patient experience of GBA-PD.

**Table 1:** Examples of open-ended questioning from the patient semi-structured interview guide

Topic	Questions
General	• Tell me about what it is like to have [GBA-PD^a^].
	• Tell me about a bad day with [GBA-PD].
	• Now tell me about a really good day for you?
	• How does your [GBA-PD] change from day to day?
	• Has your [GBA-PD] changed from one year ago to now? Please describe.
	• [If time since diagnosis is more than 1.5 years] Has your [GBA-PD] changed at all since you were first diagnosed? Please describe.
	• Does your [GBA-PD] change during the day?
Symptoms	• Tell me about any symptoms you experience that are caused by your [GBA-PD].
	• Which three of the symptoms that you described are the most bothersome for you? Why?
	• Which of the symptoms are the least bothersome for you? Why?
	• Are there any symptoms that you used to have but you don’t have any more?
	• Are there any symptoms that you are concerned about experiencing in the future?
	• Which three of those symptoms would you consider it most important for a treatment to improve? Why?
Impacts	• Tell me about how [GBA-PD] affects your daily life.
	• Are there any activities that you are now completely unable to do because of your [GBA-PD]?
	• Which impacts have the greatest effect on your life? Why?
	• Which impacts have less of an effect on your life? Why?

^a^*GBA-PD* Glucocerebrosidase-Parkinson’s Disease, during interviews, this term was replaced with the term patients’ used to describe their condition

The twenty patients took part in two-hour long, face-to-face qualitative interviews conducted by a trained interviewer. Written informed consent was obtained from all patients prior to the interview. In the interview, patients were initially asked a series of broad open-ended questions about their GBA-PD or iPD and how it affects their life, hereafter this is referred to as the ‘concept elicitation’ phase of the interview. The exploratory nature of the open-ended questions increased the chances of obtaining open and honest responses. If the patients did not spontaneously mention a concept of interest, the interviewer used more direct probes provided in the interview guide to explore these concepts directly with the patient.

Of note, the second half of both the clinician and patient interviews was dedicated to cognitive debriefing of existing measures of PD symptoms and functional impacts, to support evaluation of their relevance and content validity in a GBA-PD sample. However, the focus of this publication is on the findings from the concept elicitation section of the interview and therefore, the cognitive debriefing element of the interview is not described further.

### Qualitative analysis

All interviews were audio-recorded and transcribed verbatim and were analyzed using thematic analysis methods and Atlas.ti software [9, 10]. Participant quotes were grouped by code/theme and findings summarized and conclusions drawn. This analysis was completed by two researchers. The first transcript coded by each researcher was reviewed by a third researcher to ensure consistency. In the case of adding new codes or domains during analysis, or if there was disagreement or uncertainty about the use of codes/themes, the researchers discussed the code and reached a consensus. Participants were assigned unique ID codes to identify key information while also ensuring participant anonymity. Findings from the GBA-PD interviews were analyzed for evidence of data saturation as a means of evaluating if the sample size was sufficient, or if further interviews would elicit additional insights [11, 12]. Saturation was performed by assembling four equivalently sized groups (3 groups of 4 and one group of 3) by chronological order of interview completion and examining the elicitation patterns (spontaneous or probed) of all reported concepts. Saturation was considered to be achieved for concepts if spontaneous elicitation occurred within the first three groups.

## Results

### Demographic and clinical characteristics

Patients recruited were aged between 46 and 80 years old (GBA-PD patients were aged 52 to 80 years old). The majority of patients reported living with their spouse (*n* = 14/20, of whom 11 were GBA-PD patients) and just over half of the sample were retired (*n* = 11/20, of whom 9 were GBA-PD patients), although several patients also reported working full or part time (*n* = 7/20, of whom 5 were GBA-PD patients). Almost all of the patients were white (*n* = 18/20, of whom 15 were GBA-PD patients). Patients were at stage 1, 2, or 3 on the Hoehn and Yahr (H&Y) scale. It was not possible to recruit patients above stage 3 as the increased limitations in physical and cognitive functioning reduces patient’s ability to participate in two-hour interviews. The recruiting clinicians (*n* = 7) reported that 11 patients (including nine GBA-PD patients) had complete functional independence, seven patients (including five GBA-PD patients) had modified functional independence, whereas the remaining two (one of whom was a GBA-PD patient) required minimal assistance. Further details of the sample are included in Table 2.

**Table 2:** Demographic and clinical characteristics of GBA-PD^a^ and iPD^b^ patient samples

Demographic	Total GBA-PD^a^ (*n* = 15)	Total iPD^b^ (*n* = 5)	Total (*n* = 20)
Age, mean (range)	66.36 (52, 80)	62.60 (46, 78)	65.85 (46, 80)
Age n (%)			
46–55 years old	1 (7%)	2 (40%)	3 (15%)
56–65 years old	7 (46%)	1 (20%)	8 (40%)
66–75 years old	4 (27%)	0 (0%)	4 (20%)
76–80 years old	3 (20%)	2 (40%)	5 (25%)
Gender n (%)			
Male	8 (53%)	2 (40%)	10 (50%)
Female	7 (47%)	3 (60%)	10 (50%)
Hoehn & Yahr Scale n (%)			
Stage 1	2 (13%)	2 (40%)	4 (20%)
Stage 2	9 (60%)	2 (40%)	11 (55%)
Stage 3	4 (27%)	1 (20%)	5 (25%)
Level of functional independence n (%)			
Complete	9 (60%)	2 (40%)	11 (55%)
Modified	5 (33%)	2 (40%)	7 (35%)
Minimal assistance	1 (7%)	1 (20%)	2 (10%)
Living status n (%)			
Live alone	1 (7%)	2 (40%)	3 (15%)
Live with husband/wife	11 (73%)	3 (60%)	14 (70%)
Live with family	1 (7%)	0 (0%)	1 (5%)
Lives with caretaker	1 (7%)	0 (0%)	1 (5%)
Missing data	1 (7%)	0 (0%)	1 (5%)
Race n (%)			
White	15 (100%)	3 (60%)	18 (90%)
Black/African	0 (0%)	1 (20%)	1 (5%)
Hispanic	0 (0%)	1 (20%)	1 (5%)
Work status n (%)			
Full/part time	5 (33%)	2 (40%)	7 (35%)
Not working due to PD	0 (0%)	1 (20%)	1 (5%)
Retired	9 (60%)	2 (40%)	11 (55%)
Full time volunteer	1 (7%)	0 (0%)	1 (5%)

^a^*GBA-PD* Glucocerebrosidase Parkinson’s Disease; ^b^ iPD: idiopathic Parkinson’s Disease

The expert clinicians identified by the sponsor were based in the US, Israel and Italy. The clinicians were all neurologists or clinical geneticists and treated a mixture of GBA-PD and iPD patients. The clinicians reported that between 20 and over 200 GBA-PD patients were treated at their clinical site. The US-based clinician recruited seven of the GBA-PD patients who were interviewed as part of the study.

### Patient discussions of symptoms

Patients reported a total of 30 symptom concepts associated with their PD. A complete summary of the symptoms which were reported by 5 or more GBA-PD patients, and symptoms which patients thought were the most bothersome or important to treat is provided in Table 3. Supporting quotes for all symptom and impact concepts reported by 5 or more GBA-PD patients are included in Table 4.

**Table 3:** Patient-reported ‘most bothersome symptoms’ and ‘symptoms most important to treat’

Concept	*N* = 20 (GBA-PD)	Quote
**Symptoms**		
**Motor symptoms**		
Tremor	18 (13)	*“So when I get really scared, I really shake. And it’s embarrassing. So, so I might say [the most bothersome symptom is] an embarrassing tremor.”* (GBA-PD patient, H&Y stage 1)
Walking limitations	17 (12)	*“So when I get really scared, I really shake. And it’s embarrassing. So, so I might say [the most bothersome symptom is] an embarrassing tremor.”* (GBA-PD patient, H&Y stage 1)
Bradykinesia/ slowness	17 (12)	*“I have to do everything a lot slower than I’ve ever done it before … every year it seems to get a little worse.”* (iPD patient, H&Y stage 3)
Balance/ postural instability	16 (12)	*“I can’t be on my feet more than 15 min at a time. My balance coordination is way off.”* (iPD patient, H&Y stage 2)
Rigidity/ stiffness	16 (11)	*“What happens is the leg stiffens up. Most of the problems are in the legs right now.”* (iPD patient, H&Y stage 3)
Limb weakness	16 (11)	*“I don’t really do strenuous exercises. But if I’m going shopping sometimes, my legs get weak.”* (iPD patient, H&Y stage 2)
Speech problems	12 (11)	*“I feel it takes a little bit more effort for me to talk so that people can hear me and understand me clearly.”* (GBA-PD patient, H&Y stage 2)
**Cognitive/psychiatric symptoms**		
Memory loss	18 (13)	“*… when I talk it’s hard to talk in paragraphs. I have these long gaps in my speech. Um, there’s one now.”* (GBA-PD patient, H&Y stage 1)
Attentional impairments	14 (10)	*“I’m having problems concentrating, even on the local newspaper, which aren’t complicated articles at all, I have trouble getting to the end of it.”* (GBA-PD patient, H&Y stage 2)
**Non-motor symptoms**		
Fatigue/ tiredness	19 (14)	*“As the day progresses, I run on half battery, a quarter battery, and by 3 o’clock it’s a dead battery.”* (iPD patient, H&Y stage 2)
Pain	17 (12)	*“I’m tired of the pain, I’m always in pain, my muscles cramp, they ache.”* (iPD patient, H&Y stage 2)
Urinary problems	17 (12)	*“Oh my god, I see a urologist. I have to wear pads. I have urinary leakage. I have no control over that …*” (iPD patient, H&Y stage 2)
Orthostatic hypotension	16 (11)	*“I feel dizzy when I stand up, especially like if I’m gardening. So if I’m going to go, you know, like squatting, leaning, digging and I stand up I feel dizzy like maybe like I’m going to faint.”* (GBA-PD patient, H&Y stage 1)
Cramps	14 (10)	*“Your muscle is tightening up and it hurts like hell and it does happen every once in a while.”* (GBA-PD patient, H&Y stage 2)
Constipation	13 (9)	*“It’s getting the movement started [that is difficult] and that’s my sphincter muscle again being stiff.”* (GBA-PD patient, H&Y stage 2)
Tingling numbness	10 (9)	*“Sometimes I’m getting some tingling, numbness, like in my back.”* (GBA-PD patient, H&Y stage 2)
**Proximal motor impacts**		
Physical movement difficulties	18 (13)	“*… it’s a normal car. Just turning in that position and balancing myself to get out on my feet, that’s challenging.”* (GBA-PD patient, H&Y stage 2)
Dressing	14 (10)	*“I couldn’t do the buttons on my shirt. I just couldn’t do it, so my husband would do it for me.”* (GBA-PD patient, H&Y stage 2)
Decline in physical activities	16 (11)	*“When I first came down here, I could barely, uh, stand up in a boat to go fishing …*” (GBA-PD patient, H&Y stage 2)
Handwriting	15 (13)	*“It was difficult to read my own handwriting and it was small. And, uh, the characters were small and not as clean and smooth as I normally would have written.”* (GBA-PD patient, H&Y stage 3)
Dyskinesia	14 (10)	*“I felt that sick from the dyskinesia … Your body … is pulling and moving and hurting. So yeah, it could be painful.”* (GBA-PD patient, H&Y stage 2)
Eating and drinking	12 (9)	“*… because of the loss of smell, food doesn’t taste as good, even water, and it’s gotten worse. As time goes on, it’s harder and harder for me to find something that I like … So I’ve lost, I’ve lost weight.”* (GBA-PD patient, H&Y stage 3)
Hygiene and grooming tasks	12 (8)	*“Before I had Parkinson’s, I would take a shower every day. Now, um, I’m getting that I take a shower every other day, but I only do my hair once a week.”* (GBA-PD patient, H&Y stage 3)
Household activities and chores	11 (9)	*“I used to be a very self-sufficient person. I am no longer self-sufficient. I don’t cook. I don’t do dishes. I don’t clean up. I really just can’t. I would put myself in jeopardy of falling. And I don’t want to do that.”* (GBA-PD patient, H&Y stage 3)
Non-physical hobbies	11 (9)	*“I mean sometimes I can’t read a book because I can’t hold it. That’s how stiff I am and painful.”* (GBA-PD patient, H&Y stage 2)
**Distal impacts**		
**Sleep problems**		
Daytime sleepiness	19 (14)	*“You go to bed tired, you wake up tired, during the day you’re tired. I’m tired now. I was tired when I got up this morning.”* (GBA-PD patient, H&Y stage 2)
Sleep disturbances	18 (13)	*“I mean I sleep very restlessly. Of course I have dreams [REM sleep disorder] all night long, which is part of Parkinson’s. And so I’m restless in my sleep.”* (GBA-PD patient, H&Y stage 2)
**Role functioning limitations**		
Work	14 (11)	*“I find myself getting tired more easily ‘til I realized I was unable to practice.”* (GBA-PD patient, H&Y stage 3)
Outings	11 (8)	*“It eats up time if I go grocery shopping … I don’t want to feel like I’m, I’m weird or handicapped or anything, so I do it … The bad news is, it takes time to do it.”* (GBA-PD patient, H&Y stage 2)
**Sleep problems**		
Social isolation	17 (12)	*“I’ve cut down a lot. I’m talking like 90%. I used to be out all the time with friends … Now I just don’t, I don’t want to do it.”* (GBA-PD patient, H&Y stage 2)
Family interactions	11 (8)	*“Um, taking family trips, I can’t do that. Um, being dependable to show up for an event, maybe yes, maybe no, depends on how I feel rather than yeah, I’ll be there.”* (GBA-PD patient, H&Y stage 2)
**Emotional functioning limitations**		
Feeling fearful	17 (12)	*“Me being scared … I’ve never been a person to be scared of anything really, but, um, realizing as I get older this is the thing that’s playing on my mind.”* (iPD patient, H&Y stage 3)
Depressed mood	14 (9)	*“Depression: I mean, you know, it’s just feeling down and … you know, why do I have Parkinson’s or why am I so tired …*” (GBA-PD patient, H&Y stage 2)
Anxious mood	13 (9)	“*… anxiety is really every day, every morning for four or five hours. And just, it’s a fairly crummy four or five hours.”* (GBA-PD patient, H&Y stage 2)
Fear of falling	13 (8)	*“Well the only thing is with the legs it, uh—the weakness in my legs, I’m afraid I’m going to fall.”* (iPD patient, H&Y stage 3)
**Medical interventions**		
Treatment impacts (excluding dyskinesia)	12 (10)	*“All day long, I’m either dosing or thinking about dosing … constantly worrying about where I’m going to be when I have to take my next dose, make sure I have water with me. You know, it’s just, it’s all consuming.”* (GBA-PD patient, H&Y stage 2)

**Table 4:** Summary of symptom and proximal impact concepts reported by ≥5 GBA-PD^a^ participants

Concept (number of GBA-PD^a^ patients who reported concept; *N* = 15)	Quote
**Symptoms**	
**Motor symptoms**	
Tremor (*n* = 13/15)	*“So when I get really scared, I really shake. And it’s embarrassing. So, so I might say [the most bothersome symptom is] an embarrassing tremor.”* (GBA-PD patient, H&Y stage 1)
Walking limitations (*n* = 12/15)	*“…mobility is more difficult with GBA, which is certainly what I'm finding.”* (GBA-PD patient, H&Y stage 2)
Bradykinesia/ slowness (*n* = 12/15)	*“I just do everything much slower than I used to”* (GBA-PD patient, H&Y stage 2)
Balance/ postural instability (*n* = 12/15)	*“I’ve had problems with balancing. I’ve worked on it a lot. Uh, I take a boxing class. It helps a lot on that.”* (GBA-PD patient, H&Y stage 2)
Rigidity/ stiffness (*n* = 11/15)	*“Well the stiffness—you’re not mobile because you’re still. So it’s just stiffness. That’s my main, my main concern, my main problem.”* (GBA-PD patient, H&Y stage 2)
Limb weakness (*n* = 11/15)	*“I would describe that as weakness, when it’s acting up, yes … it’s like you’ve overworked it for a long period of time and it’s, uh, it’s not sore but it’s just kind of irritated.”* (GBA-PD patient, H&Y stage 2)
Speech problems (*n* = 11/15)	*“I feel it takes a little bit more effort for me to talk so that people can hear me and understand me clearly.”* (GBA-PD patient, H&Y stage 2)
**Cognitive/psychiatric symptoms**	
Memory loss (*n* = 13/15)	“*… when I talk it’s hard to talk in paragraphs. I have these long gaps in my speech. Um, there’s one now.”* (GBA-PD patient, H&Y stage 1)
Attentional impairments (*n* = 10/15)	*“I’m having problems concentrating, even on the local newspaper, which aren’t complicated articles at all, I have trouble getting to the end of it.”* (GBA-PD patient, H&Y stage 2)
REM^b^ sleep disorder (*n* = 7/15)	*“I act out my dreams. Usually my wife tells me they’re sports dreams. She occasionally gets whacked.”* (GBA-PD patient, H&Y stage 1)
Apathy (*n* = 6/15)	*“It’s almost like I lose my ambition to get something done … The sense of accomplishment is just like, I don’t care.”* (GBA patient, H&Y stage 3)
**Non-motor symptoms**	
Fatigue/ tiredness (*n* = 14/15)	*“I have unexplained tiredness, uh, about 1:00 in the afternoon, something like that. And that can come on quite suddenly.”* (GBA-PD patient, H&Y stage 2)
Pain (*n* = 12/15)	*“Like a stiffness pain. You know, that was sort of going on with the stiffness. Like my hand was stiff and almost like a—maybe like a tendonitis pain like just pulling.”* (GBA-PD patient, H&Y stage 1)
Urinary problems (*n* = 12/15)	*“I’m constantly—in fact, that’s why I had to run to the bathroom when I got here. I have urges of frequency.”* (GBA-PD patient, H&Y stage 3)
Orthostatic hypotension (*n* = 11/15)	*“I feel dizzy when I stand up, especially like if I’m gardening. So if I’m going to go, you know, like squatting, leaning, digging and I stand up I feel dizzy like maybe like I’m going to faint.”* (GBA-PD patient, H&Y stage 1)
Cramps (*n* = 10/15)	*“Your muscle is tightening up and it hurts like hell and it does happen every once in a while.”* (GBA-PD patient, H&Y stage 2)
Constipation (*n* = 9/15)	*“It’s getting the movement started [that is difficult] and that’s my sphincter muscle again being stiff.”* (GBA-PD patient, H&Y stage 2)
Tingling numbness (*n* = 9/15)	*“Sometimes I’m getting some tingling, numbness, like in my back.”* (GBA-PD patient, H&Y stage 2)
Sweating/ oily skin (*n* = 6/15)	*“I’ll get white and sweaty and then I’m like, okay it’s time for either a dose or for me to sit down.”* (GBA-PD patient, H&Y stage 3)
**Proximal motor impacts**	
Physical movement difficulties (*n* = 13/15)	“*… it’s a normal car. Just turning in that position and balancing myself to get out on my feet, that’s challenging.”* (GBA-PD patient, H&Y stage 2)
Handwriting (*n* = 13/15)	*“It was difficult to read my own handwriting and it was small. And, uh, the characters were small and not as clean and smooth as I normally would have written.”* (GBA-PD patient, H&Y stage 3)
Decline in physical activities (*n* = 11/15)	*“When I first came down here, I could barely, uh, stand up in a boat to go fishing …*” (GBA-PD patient, H&Y stage 2)
Dressing (*n* = 10/15)	*“I couldn’t do the buttons on my shirt. I just couldn’t do it, so my husband would do it for me.”* (GBA-PD patient, H&Y stage 2)
Dyskinesia (*n* = 10/15)	*“I felt that sick from the dyskinesia … Your body … is pulling and moving and hurting. So yeah, it could be painful.”* (GBA-PD patient, H&Y stage 2)
Eating and drinking (*n* = 9/15)	“*… because of the loss of smell, food doesn’t taste as good, even water, and it’s gotten worse. As time goes on, it’s harder and harder for me to find something that I like … So I’ve lost, I’ve lost weight.”* (GBA-PD patient, H&Y stage 3)
Household activities and chores (*n* = 9/15)	*“I used to be a very self-sufficient person. I am no longer self-sufficient. I don’t cook. I don’t do dishes. I don’t clean up. I really just can’t. I would put myself in jeopardy of falling. And I don’t want to do that.”* (GBA-PD patient, H&Y stage 3)
Non-physical hobbies (*n* = 9/15)	*“I mean sometimes I can’t read a book because I can’t hold it. That’s how stiff I am and painful.”* (GBA-PD patient, H&Y stage 2)
Hygiene and grooming tasks (*n* = 8/15)	*“Before I had Parkinson’s, I would take a shower every day. Now, um, I’m getting that I take a shower every other day, but I only do my hair once a week.”* (GBA-PD patient, H&Y stage 3)
Limb agility (*n* = 7/15)	*“I’m trying to tap my leg. I’m fighting through something … when I went to the first neurologist and he asked me to do that I was like I, I’m surprised that I can’t do it.”* (GBA-PD patient, H&Y stage 1)
Decrease in activities (*n* = 6/15)	*“Planning is, is a huge part of, um, having Parkinson’s because I know my best time of the day is in the morning. So I have to accommodate trying to get everything done in that timeframe because by 2:00 … my body says you’re done.”* (GBA-PD patient, H&Y stage 3)
**Distal impacts**	
**Sleep problems**	
Daytime sleepiness (*n* = 14/15)	*“You go to bed tired, you wake up tired, during the day you’re tired. I’m tired now. I was tired when I got up this morning.”* (GBA-PD patient, H&Y stage 2)
Sleep disruptions (*n* = 13/15)	*“I mean I sleep very restlessly. Of course I have dreams [REM sleep disorder] all night long, which is part of Parkinson’s. And so I’m restless in my sleep.”* (GBA-PD patient, H&Y stage 2)
**Role functioning limitations**	
Work (*n* = 11/15)	*“I find myself getting tired more easily ‘til I realized I was unable to practice.”* (GBA-PD patient, H&Y stage 3)
Outings (*n* = 8/15)	*“It eats up time if I go grocery shopping … I don’t want to feel like I’m, I’m weird or handicapped or anything, so I do it … The bad news is, it takes time to do it.”* (GBA-PD patient, H&Y stage 2)
**Social functioning limitations**	
Social isolation (*n* = 12/15)	*“I’ve cut down a lot. I’m talking like 90%. I used to be out all the time with friends … Now I just don’t, I don’t want to do it.”* (GBA-PD patient, H&Y stage 2)
Family interactions (*n* = 8/15)	*“Um, taking family trips, I can’t do that. Um, being dependable to show up for an event, maybe yes, maybe no, depends on how I feel rather than yeah, I’ll be there.”* (GBA-PD patient, H&Y stage 2)
**Emotional functioning limitations**	
Feeling fearful (*n* = 12/15)	*“I have more recently felt some feelings of panic, um, because I maybe couldn’t do—function with something or I couldn’t remember it or …*” (GBA-PD patient, H&Y stage 2)
Depressed mood (*n* = 9/15)	*“Depression: I mean, you know, it’s just feeling down and … you know, why do I have Parkinson’s or why am I so tired …*” (GBA-PD patient, H&Y stage 2)
Anxious mood (*n* = 9/15)	“*… anxiety is really every day, every morning for four or five hours. And just, it’s a fairly crummy four or five hours.”* (GBA-PD patient, H&Y stage 2)
Fear of falling (*n* = 8/15)	*“I don’t cook. I don’t do dishes. I don’t clean up. I really just can’t. I would put myself in jeopardy of falling.”* (GBA-PD patient, H&Y stage 3)
Feeling upset (*n* = 8/15)	“*… first it’s first finding about it [the diagnosis], which is very devastating.”* (GBA-PD patient, H&Y stage 2)
Embarrassment (*n* = 6/15)	*“The worst thing, um, if it’s a bad day and you’re moving around a lot, it’s embarrassing.”* (GBA-PD patient, H&Y stage 2)
Stress (*n* = 6/15)	*“I mean I do get a little tense if I’m with a group of people on a given day. If I’m by myself I’m okay.”* (GBA-PD patient, H&Y stage 2)
**Medical interventions**	
Treatment impacts (excluding dyskinesia; *n* = 10/15)	*“All day long, I’m either dosing or thinking about dosing … constantly worrying about where I’m going to be when I have to take my next dose, make sure I have water with me. You know, it’s just, it’s all consuming.”* (GBA-PD patient, H&Y stage 2)

^a^*GBA-PD* Glucocerebrosidase-Parkinson’s Disease, ^b^*REM* Rapid Eye Movement

With the exception of rapid eye movement (REM) sleep disorder and dry mouth, all symptoms reported by five or more patients were reported by both GBA-PD patients and iPD patients, and discussed in a similar manner by both groups. REM sleep disorder was reported by seven GBA-PD patients, one of whom described the symptom as the most important to treat.

*“Oh, the worst part of it. The, um, vivid dreams and screaming in my sleep.”* (GBA-PD patient, H&Y stage 3)

The majority of symptoms were reported by GBA-PD and iPD patients from all three stages on the H&Y scale represented in this study, but for most symptoms severity broadly seemed to increase with H&Y stage, as would be expected. The exceptions were excessive drooling/saliva, difficulty chewing or swallowing, apathy, excessive sweating, and dry mouth which were all reported by only patients in stages 2 and 3 on the H&Y scale, suggesting that they are symptoms not typically present in early PD. Unsurprisingly, these symptom concepts were reported by less than 10 GBA-PD patients. Four symptoms were consistently reported to be the most impactful and the most important to treat for GBA-PD patients. Detailed summaries of each of these symptoms are provided below.

**Tremors** were reported to occur in the hands (*n* = 11), legs (*n* = 4), arms (*n* = 2), and/or shoulder (n = 1), of GBA-PD patients. They were consistently referred to as ‘tremors’ or ‘shaking’. Tremors had a major impact on patient’s lives, and were considered one of the most bothersome symptoms by five patients, and one of the most important symptoms to treat by four patients. Tremors were reported to occur for a range of different durations and also varied widely across the sample in terms of how often they occurred.

*“There was just a small rotation, uh, that was constant in the left hand, uh, shaking up and down I guess is the best way to describe it.”* (GBA-PD patient, H&Y stage 3)

**Rigidity/stiffness** was reported to occur predominantly in the legs (*n* = 4) and arms (n = 4) of GBA-PD patients. The term ‘stiffness’ was most commonly used to describe the symptom, and patients also described the stiffness as feeling like ‘a tight rubber band’. Patients often discussed stiffness and pain in conjunction, and noted that combined, these symptoms impacted their HRQoL. Rigidity/stiffness was considered one of the most bothersome symptoms by three GBA-PD patients.

*“I'm really stiff. The bad days are becoming more, more numerous. And I'm just stiff and in pain”* (GBA-PD patient, H&Y stage 2)

**Speech problems** reported by GBA-PD patients included: their voice getting softer/quieter (*n* = 7), difficulty pronouncing or enunciating words (*n* = 4) and speaking too slow or too fast (*n* = 3). Some patients reported multiple changes in their speech. These changes could sometimes be resolved with speech therapy, or when patients specifically focussed on their speech during discussions. Speech problems were frustrating to patients, as they made it difficult to communicate. This led to four GBA-PD patients listing this symptom as one of the most bothersome and two GBA-PD patients listing this symptom as being one of the most important to treat.

*“I try to speak loud and slowly. If I can’t do that, I start slurring like crazy … which makes it hard to speak.”* (GBA-PD patient, H&Y stage 3)

**Memory loss** that GBA-PD patients reported included a decline in verbal fluency (*n* = 11), which impacted their ability to have fluent conversations, and difficulty remembering things in everyday life (*n* = 3), such as where they left keys. Cognitive decline was considered very concerning as patients associated it with a loss of self. Three GBA-PD patients listed this symptom as one of the most bothersome and two patients listed this symptom as being one of the most important to treat.

*“Now I'm losing my thoughts here, which is another thing that has affected me … I feel like I'm having memory loss.”* (GBA-PD patient, H&Y stage 2)

*“Well I worry about further decline, cognitive decline … If I don’t have my mind, I'm not me at all.”* (GBA-PD patient, H&Y stage 1)

### Patient discussion of impacts

**Fig. 1:**
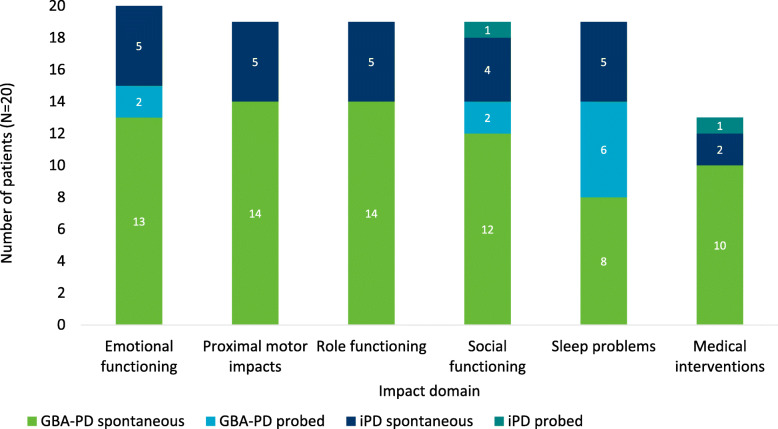
Impact domains reported by patients

Six impact domains were reported by GBA-PD patients (Fig. 1); these were categorised into one proximal motor impact domain (*n* = 14) and the five distal impact domains of: emotional functioning (*n* = 15), role functioning (*n* = 14), social functioning (*n* = 14), sleep problems (*n* = 14), and medical interventions (*n* = 10). Concepts were categorized as impacts based on the ways in which patients characterised the concept as being the result of a symptom. All impact domains were reported by patients in all three H&Y stages represented in this study, although patients did vary in the severity of the impacts, broadly in line with their H&Y stage. All impact domains reported by GBA-PD patients were also reported by the iPD patients in the study. Supporting quotes for all impact concepts reported by 5 or more GBA-PD patients are included in Table 4.

**Proximal motor impacts** included key proximal characteristics of PD which were not directly symptoms of PD, and which produced other distal impacts and impacted HRQoL. Impact concepts included in the proximal motor impact domain include: handwriting impairment (*n* = 13), physical movement difficulties (*n* = 15), and dyskinesia (which is recognised to be a side effect of treatment) (*n* = 10).

*“ … your body is just like reacting to the medicine. So my toe is curling. My left ankle is twisting. You know, I'm moving. I hate that.”* (GBA-PD patient, H&Y stage 2)

*“It was difficult to read my own handwriting and it was small. And, the characters were small and not as clean and smooth as I normally would have written.”* (GBA-PD patient, H&Y stage 3)

**Emotional functioning** impacts were experienced by all 15 GBA-PD patients. The emotional impact most commonly reported by GBA-PD patients was fear (*n* = 12), including most commonly a fear of falling (*n* = 8). Other emotional impacts frequently reported by GBA-PD patients include depression (*n* = 9) and anxiety (*n* = 9). These emotional impacts were most commonly discussed as being a result of the PD diagnosis but were also noted to be due the experience of symptoms or other impacts.

*“Parkinson's patients typically have balance issues and often fall. So I’m always aware of that … I don’t want to fall if I get on a chair … So I’m cautious, cautiously active.”* (GBA-PD patient, H&Y stage 2)

*“It's depressing. Yes, it is. And mostly because … you don’t know how much worse it's going to get and when.”* (GBA-PD patient, H&Y stage 2)

**Role functioning** impacts reported by GBA-PD patients included limited ability to do work (*n* = 11), leave the house (*n* = 8) or be independent (*n* = 3). These impacts were attributed to a variety of motor and non-motor symptoms including attentional impairments, fatigue/tiredness, and tremor.

*“It was caused by the fact that I couldn’t write well enough in my lab notebooks and I couldn’t concentrate enough to, uh, to run … essentially a high technology company. And, uh, I had to sell the company.”* (GBA-PD patient, H&Y stage 2)

**Social functioning** impacts included reduced amounts of socializing or changes in social dynamics (*n* = 12) and altered family relationships (*n* = 8). Symptoms impacting the ability to hold conversations (such as memory loss and speech problems), embarrassment about tremors or urinary incontinence, and worry about the future were among the concepts reported to impact social functioning.

*“I'm already having a hard time putting together the words I want to say to somebody … if I'm in a gather of more than like three or four people I won't, I won't talk because I feel like I have to fight to get—and I'm like, no, it's not worth it.”* (GBA-PD patient, H&Y stage 3)

**Sleep problems** reported included daytime sleepiness (*n* = 14), and sleep disruptions (*n* = 13). Daytime sleepiness was most commonly associated with tiredness/fatigue while sleep disruptions at night were reported to be due to a range of symptoms including REM sleep disorder (*n* = 3), pain (*n* = 2), urinary problems (*n* = 2), and tremors (*n* = 2).

*“ … last night I woke up four times to go to the bathroom … And when I, ah, try to go back to sleep after I wake up, then I have tremors.”* (GBA-PD patient, H&Y stage 2)

**Medical interventions** reported included impacts of taking treatment (excluding dyskinesia) (*n* = 8) and disruptive doctor’s appointments (*n* = 5).

*“I don’t like taking medicine because it gives you other side effect … ”* (GBA-PD patient, H&Y stage 2)

The impact domains and concepts reported by GBA-PD patients were also all reported without notable differences by the iPD patients in the study.

### Conceptual saturation

Twenty-five out of the 30 symptoms reported by GBA-PD patients were spontaneously reported for the first time in the first three saturation groups (each containing *n* = 4/15 participants). Sweating/oily skin (reported by *n* = 7/15 patients, *n* = 2 spontaneously) was spontaneously reported in the final saturation group (containing *n* = 3/15 participants). The remaining four symptom concepts were only elicited when probed, were all reported by less than half the GBA-PD sample, and included: cramps (*n* = 6/15), constipation (*n* = 5/15), hallucinations/psychosis (*n* = 5/15), and pain (*n* = 3/15). These symptoms, although not mentioned spontaneously within the first three groups of patients, were all thoroughly discussed when probed and thus are considered to have been adequately explored.

In the GBA-PD sample, all impact concepts (total 33) were elicited spontaneously within the first three saturation groups.

The findings from the saturation analysis indicate that a sample of 15 GBA-PD participants was adequate to achieve concept saturation of the majority of symptom and impact concepts.

### Expert clinician discussion of symptoms and impacts

The expert clinicians confirmed that the symptom and impact concepts in the draft conceptual model were all relevant for GBA-PD patients. Specifically, clinicians confirmed that all of the most important symptoms and impacts (tremor, memory loss, rigidity/stiffness, and speech problems) were experienced by the patients that they treated with GBA-PD. Clinicians also identified REM sleep disorder and a decline in visual acuity as symptoms of GBA-PD. REM sleep disorder was only reported by GBA-PD patients in the sample; however, a decline in visual acuity was reported by both GBA-PD and iPD patients. The alignment between the findings from the clinician interviews and the GBA-PD patient interviews provides support for the generalizability of the results of this study.

Further, the clinicians commented that GBA-PD and iPD patients’ disease experience is broadly similar. However, clinicians (*n* = 4/5) agreed that cognitive/psychiatric symptoms might present earlier and progress more rapidly in GBA-PD patients, as compared to iPD patients.

### Updated GBA-PD conceptual model

Based on findings from the GBA-PD patient and clinician interviews the draft conceptual model was updated. The updated conceptual model included additional concepts which were spontaneously discussed during the patient interviews such as REM sleep disorder (this and other symptoms were also spontaneously reported during clinician interviews). Additionally, more nuance was added to concepts which had previously been included, and some concept names were adjusted to use language that reflected the patient language. For example: cramps, tingling/numbness, and dystonia were included as separate concepts rather than grouped together as one ‘other sensations’ concept. In addition, ‘rigidity’ was reworded to ‘rigidity/stiffness’, to reflect how patients described the concept while also capturing the medical terminology for the symptom.

In the updated conceptual model, the organization of concepts as symptoms and impacts was also adjusted based on the ways in which patients discussed different concepts during interviews. For example, depressed mood and anxious mood were redefined as impacts rather than symptoms, as during the interviews they were discussed as occurring as a consequence of PD symptoms or proximal impacts. When rearranging concepts, a proximal motor impacts domain was introduced to include the impacts which were described as being most closely related to symptoms and which led to further, more distal impacts on other aspects of life and overall patient HRQoL.

The updated conceptual model also included adjustments to the relative prevalence of symptoms in GBA-PD and iPD patients. Based on the clinician and patient interviews, the only category which might be considered more prevalent in the GBA-PD population was cognitive/psychiatric symptoms; and only patients with GBA-PD reported REM sleep disorder. No other differences between GBA-PD and iPD are highlighted in the conceptual model as, based on this small, qualitative study, there was no clear, concrete evidence of other differences.

**Fig. 2:**
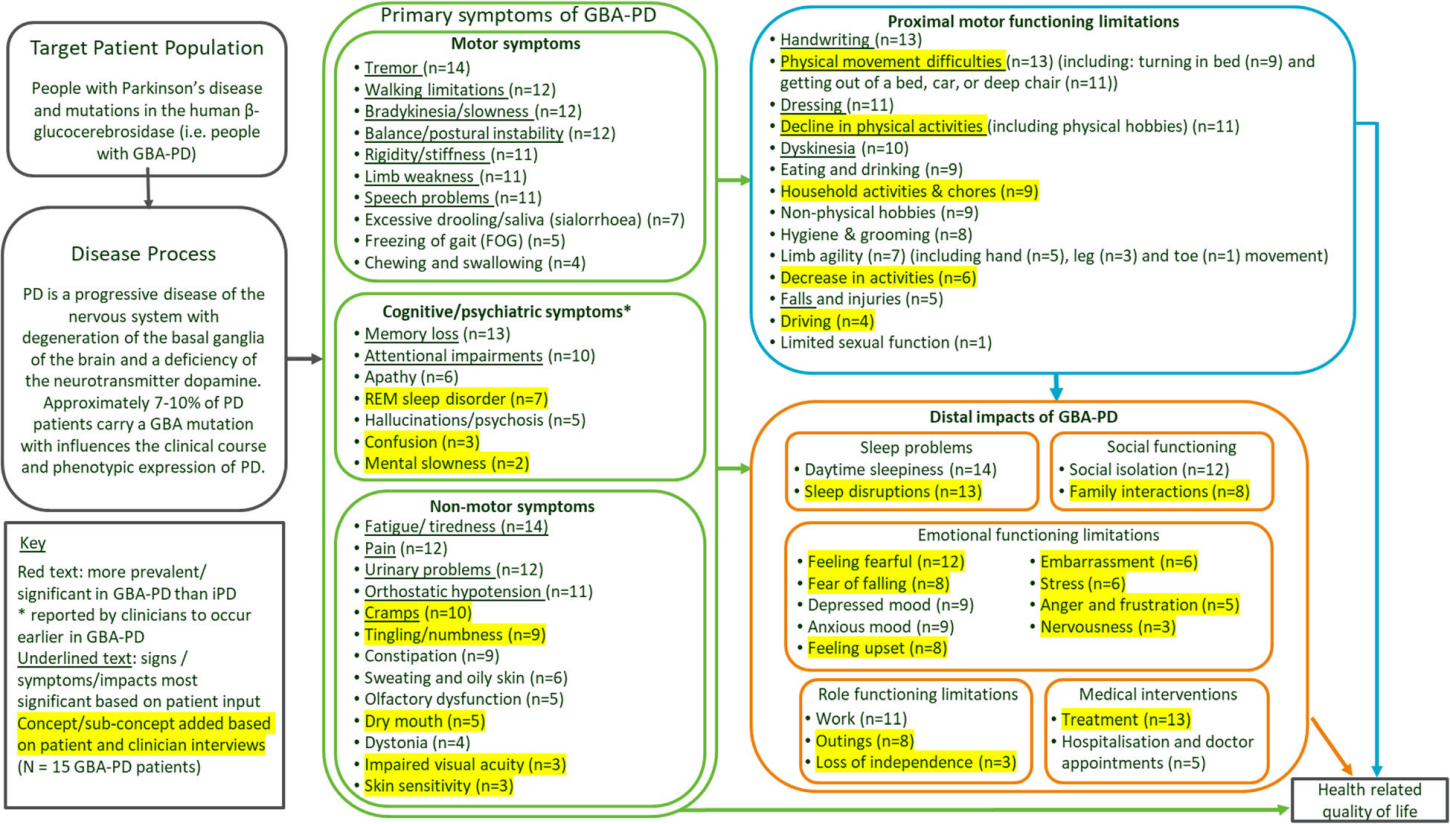
Conceptual model of GBA-PD

The conceptual model (Fig. 2) is based on the GBA-PD patient experience; and so includes the number of GBA-PD patients who reported each concept.

## Discussion

Throughout the interviews, GBA-PD patients predominantly focussed on motor symptoms, which aligns with the findings from qualitative literature on PD patients [5]. While over 30 symptoms concepts were reported, those which were reported to be the most important and impactful were: tremor, rigidity/stiffness, speech problems and memory loss. These concepts are included within the most important domains (motor symptoms and cognitive impairment) reported in previous qualitative research with iPD patients [5]. Other important and commonly reported concepts include: fatigue/tiredness, walking limitations, slowness/bradykinesia, balance/postural instability, pain, urinary problems, limb weakness, attentional impairments, orthostatic hypotension, cramps, and several proximal motor functioning limitations (namely: handwriting, physical movement limitations, dressing, decline in physical activities, and dyskinesia).

The symptom and impact concepts elicited from the GBA-PD patient interviews broadly aligned with those reported in existing quantitative literature [3, 4] on the GBA-PD patient experience and in qualitative research on iPD patients [5]. Additionally, the presence of excessive drooling/saliva and difficulty chewing or swallowing only in patients at H&Y stage 2 or 3 is echoed in resources for advanced PD patients [13]. The findings from the patient interviews were also consistent with the feedback provided by the expert clinicians interviewed. While patient interview findings aligned with previous research and clinician perspective, the patient findings also provided a more in-depth understanding of the GBA-PD patient-experience of each concept and provided evidence to support breaking some of the higher-level domains down into more specific concepts. For example, ‘sleep problems’ were divided to include ‘sleep disturbances’ and ‘REM sleep disorder’; and ‘cognitive impairments’ were adjusted to include ‘memory loss’, ‘attentional impairments’, ‘confusion’, and ‘mental slowness’. The more detailed understanding of concepts associated with GBA-PD adds to the existing literature and allows a more nuanced and accurate representation of the patient experience.

The findings confirm that the concepts relevant to assess in GBA-PD patients are consistent with those relevant to assess in iPD patients. These findings broadly align with previous quantitative research [14–16] suggesting that the presentation of GBA-PD and iPD is largely similar. Expert clinicians, who had experience with a wider population of PD patients also reported that the GBA-PD and iPD patient experiences are predominantly similar. Given the small sample of iPD patients in this qualitative research, it is not possible to draw firm conclusions regarding the relative prevalence of most symptoms mentioned. However, the tentative conclusion that REM sleep disorder is more prevalent in the GBA-PD population (based on this symptom only being reported by GBA-PD patients in this study) is supported by previously published quantitative research that indicates patients with a GBA mutation were significantly more likely than iPD patients to experience REM sleep disorder (*p* = 0.039) [17]. Additionally, expert clinician feedback provided preliminary evidence of a difference between the GBA-PD and iPD populations in terms of the cognitive and psychiatric symptoms domain. It is not possible to make further claims relating to symptom prevalence due to small iPD sample.

The sample of iPD patients in this study was very small, as it was not the original intention to include those patients. Due to the small sample size, findings related to the iPD patient experience have been used as a reference point for the findings from GBA-PD patient interviews. The findings from both GBA-PD and iPD interviews are consistent with the literature and do provide support for consistency in the disease experiences of GBA-PD and iPD patients; however, due to the small iPD sample size they should be generalized with caution. Given the limited amount of available qualitative research in GBA-PD patients, the interview guides were developed in part from literature in the iPD population. While the probed questions in the guide did therefore presuppose similarities between iPD and GBA-PD, many of these concepts were spontaneously mentioned by the GBA-PD patients.

In terms of study limitations, the study sample was predominantly white and patients were only interviewed in the US. Given evidence of variation in symptom presentation between different ethnicities [18], further study in patients of other races and countries/cultures is recommended to confirm that the findings are generalisable. Further this study only includes patients at H&Y stage 1 to 3, and as such the results are not generalizable to patients at other stages. Additional study in later stage PD patients would also ideally be conducted, but the inherent challenge of recruiting and interviewing patients with advanced PD must be recognised. It is likely to be even more difficult to recruit patients with GBA-PD in an advanced stage of the disease due to the rarity of the confirmation of the GBA mutation, and the evidence of more rapid cognitive decline in this population. The challenge of interviewing late-stage patients could perhaps be compensated for by also interviewing caregivers of more advanced patients. Such caregiver research is also recommended in general to add a further perspective to improve understanding of the patient experience of GBA-PD.

## Conclusion

The findings confirm that the concepts relevant to assess in GBA-PD patients are consistent with those relevant to assess in iPD patients. The updated conceptual model developed based on these findings can be used to guide the selection of key measurement concepts for assessment in future clinical trials for GBA-PD and as a basis for understanding the patient experience of this condition. Further studies to validate the questionnaires which were debriefed as part of this study (The Movement Disorder Society-United Parkinson’s Disease Rating Scale and The Parkinson’s Disease - Cognitive Rating Scale) will be conducted to validate these studies in a larger sample.

## Data Availability

Not applicable.

## Abbreviations

GBA: Glucocerebrosidase
H&Y: Hoehn & Yahr
HRQoL: Health related quality of Life
IPD: Idiopathic Parkinson’s disease
PD: Parkinson’s Disease
REM: Rapid eye movement
